# The bachelor of medicine and bachelor of surgery program for international students in China: policies, assessments and challenges

**DOI:** 10.3389/fmed.2025.1553628

**Published:** 2025-04-10

**Authors:** Zheng Huang

**Affiliations:** Key Laboratory of Environment and Health, Ministry of Education and Ministry of Environmental Protection, and State Key Laboratory of Environmental Health, School of Public Health, Tongji Medical College, Huazhong University of Science and Technology, Wuhan, China

**Keywords:** IoME, MBBS program, China, policy, assessment, challenge

## Abstract

Internationalization of medical education (IoME) in China is an integral part of the broader higher education internationalization efforts, a process that has been strategically designed and implemented by the Chinese government since the introduction of the opening-up policy in the late 1970s. The bachelor of medicine and bachelor of surgery (MBBS) program, a 6-year English medium undergraduate program with an international curriculum for overseas medical students, provides a new perspective on the IoME in China. This paper expounds on the national policy development of the IoME and MBBS program, summarizes the assessments of the program, and highlights the challenges arising from students (e.g., selection and support), curricula (e.g., curriculum integration) and faculties (e.g., faculty improvement) that constitute the core of the 3-dimensional model of the IoME. Improving the quality of the MBBS program would contribute to building a brand project of the IoME in China and balancing the medical workforce supply at the regional and global levels.

## 1 Introduction

A widely accepted definition of the internationalization of medical education (IoME) was provided by Wu et al., as the “*process of purposefully integrating international, intercultural, or global dimensions into medical education in order to enhance its quality and prepare graduates for professional practice in a globalized world*” (1).

IoME has become increasingly important because of the international health crisis, such as the COVID-19 pandemic, and the distinctive disease spectrum in the cross-border context placed new requirements for equipping the medical graduates with global skills to carry out healthcare in various cultural settings (1, 2). Moreover, the migration of healthcare workers, particularly from low- and middle-income countries to high-income countries, would lead to a shortage of skilled professionals and reduce the quality of care available to the population in the source countries (3). IoME also presents a potential avenue for alleviating the shortage of health professionals in addition to enhancing capacity building in exporting countries (4). The integration of IoME could be encouraged by several factors, such as the globalization of healthcare, government pressure, and competitiveness between higher education institutions (HEIs), and could be achieved with the 3-dimensional model based on the student (local or international), the teacher (local or international), and the curriculum (local, imported, or international) (5). Each HEI could choose innovative pathways to the IoME on the basis of its own strategy, strength, and resources.

Through continuous efforts in the past 70 decades, China has gradually built an extensive medical education system to meet the ever-increasing demand for health care for 1.4 billion people (6, 7). In 2022, 333,657, 81,388, and 14,819 students graduated from this medical education system with bachelor’s, master’s, and doctoral degrees, respectively (8). There have been tailored attempts in some Chinese HEIs aimed at enhancing the level of the IoME for local students. These include curriculum reform, improvements in pedagogy, and the establishment of joint programs with foreign HEIs (9–11). Meanwhile, there is a unique medical undergraduate program offered by Chinese HEIs, a 6-year English medium program for international medical students (IMSs), who are conferred with a bachelor of medicine and bachelor of surgery (MBBS) on successful completion of their studies. Established in 2007, this program provides an exclusive perspective on the IoME in China through the lens of foreign student admission, curriculum reformation, and faculty optimization. Consequently, China has become the preferred destination for medical education, particularly for IMSs from developing countries (12).

National policies for the internationalization of higher education (IoHE) in China lay the foundation for the inception and development of the IoME and MBBS program; nonetheless, there is a paucity of research on the development of those policies. Over the past dozen years, several Chinese and English papers have investigated the performance of the MBBS program in China, mostly from the perspective of IMSs during their undergraduate studies (13–15), and limited available data on post-graduation tracking have been neglected. This paper aims to review the development of the IoME in China, particularly focusing on the MBBS program for IMSs. It first outlines the national policy development of the IoME and MBBS program, encompassing policy formulation and the transition from a focus on quantity to an emphasis on quality. The paper then summarizes perspectives on the evaluation of the MBBS program based on existing research targeting IMSs both before and after graduation. Finally, it delves into the challenges associated with enhancing the performance of the MBBS program and offers actionable recommendations to address these issues. It is anticipated to offer valuable insights for the sustainable and steady development of the MBBS program in China.

## 2 Policies development of the IoME and MBBS program

In China, the IoME is embedded in the development of the IoHE, which, led by the government acting as a policy maker (16), took off after the opening-up policy was implemented in 1978 (16, 17), hosting 1,236 international students in that year. The policy leads China to open its doors to the outside world, enabling the country to learn new knowledge and adopt positive experiences from other countries. It blends these insights with its own cultural traditions to invigorate China’s economy and enhance its science and technology capabilities (18).

In 1980, the *Regulations of the People’s Republic of China on Academic Degree* (19) entitled HEIs to grant degrees to international students who attained acquired academic levels. Further reforms came in 1989 with the announcement of the *Regulations on Enrollment of Self-financed International Students*, which granted HEIs greater autonomy in admitting overseas students (20). As a result, the proportion of self-funded international students among all international students increased dramatically, rising from 50% in 1990 to 90% by 2000. In 2001, China joined the World Trade Organization (WTO) and released the *10th Five-Year Plan for Educational Development* (21), which insisted on the steady expansion of international students, and became a major player in the global arena of international student mobility. In 2010, the Chinese Ministry of Education (MOE) launched the *Study in China Plan* (22), an initiative aimed at enhancing the IoHE in China. This plan laid out a comprehensive framework for admission policies, branding of academic programs and courses, faculty development, and quality assurance systems. As a result of these efforts, by 2018, China had enrolled 492,185 international students from 196 different countries and regions, placing it as the third most popular destination for international students, just behind the United States and the United Kingdom.

Several academics have also put forward recommendations for the formulation and implementation of policies on the IoHE, such as:

- Improving the international education support system, including academic degree mutual recognition, graduates’ employability, alumni’s connection, a system for monitoring and evaluating the quality of performance, and capacity-building (23–25).
- Gradually introducing market-based mechanisms or other approaches beyond government-led schemes, including education marketing strategy, diversified scholarship sources and funding systems, and online and outreach promotion (23, 24).

The number of IMSs pursuing western medicine education has also increased rapidly since the new millennium, from 1,626 in 2001 to 9,605 in 2005, and quality assurance has become increasingly prominent due to the uneven development of medical colleges and universities. In 2005, an expert panel, namely the National Working Group of Experts on Education for International Students Majoring in Medicine (Western Medicine), was appointed by the MOE to develop regulations on quality standards for the MBBS program. The *Interim Provisions for Quality Control Standards on Undergraduate Medical Education in English for International Students in China* (26) was issued in 2007, indicating the official establishment of the MBBS program in China. It was the first account of basic norms for the MBBS program, and was set by referencing “WHO guidelines for quality assurance of basic medical education in the western Pacific region” (27), “Global minimum essential requirements in medical education” (28), “Basic medical education. WFME global standards for Quality Improvement” (29), and “Standards for basic medical education in China (trial)” (30). This regulation is comprised of 7 chapters and a total of 32 articles, providing comprehensive guidelines for IMS recruitment, enrollment, curriculum, training, teaching and supervision, and basic HEI conditions for the MBBS program.

The regulation specifies that the curriculum consists of three main parts:

- Chinese culture and natural science courses, such as the Chinese language, chemistry, mathematics.
- basic medical sciences, behavioral science, preventive medicine, and clinical medicine, covering disciplines including anatomy, physiology, biochemistry, microbiology, pathology, immunology, psychology, medical ethics, community health, diagnosis, internal medicine, surgery, obstetrics and gynecology, pediatrics.
- Internship.

In general, the first year of study includes courses in liberal arts and natural sciences. This initial phase is followed by 2 years of preclinical studies, which focus on basic medical sciences. During the fourth and fifth years, students engage in clinical courses integrated with bedside teaching. The final, or sixth, year is dedicated to rotational internships (13, 31, 32).

The approved HEIs and allocated seats for the MBBS program were released annually by the MOE since 2007 based on an expert survey on program performance through lecture observation, discussion with IMSs, access to administration documents, and observation of clinical placement and rotation internships. In the 2023/2024 academic year, 44 HEIs were authorized to enroll a total of 3,048 freshmen in the MBBS program.

Under the guidance and support of the MOE, the China Education Association for International Exchange (CEAIE) engaged in quality assurance programs as a third-party certification organization. CEAIE issued quality accreditation rules and standards for the MBBS program in 2021 (33, 34), and on-site inspections were carried out by CEAIE for the first five HEIs in December, 2023.

Put succinctly, the IoME and MBBS policy focus witnessed a shift from quantity expansion to quality control, and was integrated in an endeavor to augment the global image, reputation and market appeal of Chinese HEIs.

## 3 Assessments of the MBBS program

In the past 10 years, there have been a handful of studies on assessments of the MBBS program performance in China, mostly based on questionnaire surveys among IMSs before graduation.

The majority of the 209 final-year IMSs from 4 universities located in Xuzhou, Dali, Nanjing and Haikou were satisfied with the quality of medical education (76.1%) and the clinical relevance of basic science coursework (70.8%). They believed that they had acquired sufficient clinical skills to begin a residency program (71.3%), and oral Chinese skills were critical for the effectiveness of clinical clerkships (13). Seventy-four percent of the respondents at a university located in Shanghai considered the MBBS course setting to be appropriate or neutral, and 1/3 reported language barriers to be the greatest difficulty in clinical practice (14). A 4-point Likert scale (excellent, good, fair, poor) was adopted to measure the quality of MBBS courses by 98 five-grade IMSs in a Hainan-based university. The percentage of “excellent” or “good” courses varied from 54 to 89% among 15 basic medical and clinical courses, and more than half of the IMSs thought teachers should improve their English proficiency (15). Researchers at a Kunming-based university investigated the evaluation of 153 fifth- and sixth-grade IMSs for course teachers and clinical clerkships by a 3-point Likert scale (highly satisfactory, basically satisfactory, or unsatisfactory). Most of the respondents were highly or basically satisfied with teachers’ management (83.2%), enthusiasm (80.4%) and question feedback (75.2%) during course delivery. In contrast, a sizable percentage of students were unsatisfied with medical history (49.7%) and operation opportunities (60.6%) during clinical clerkships (35).

A qualitative study of 2 universities involving 40 IMSs revealed that support systems and campus resources are positive factors for academic success, while language barriers, curriculum differences between China and its home countries, and internship difficulties negatively affect the MBBS program outcomes. Furthermore, the bar needs to be raised for the admission of IMSs, both in terms of linguistic and academic ability (36).

There are limited available data on the post-graduation follow-up among the MBBS graduates. A total of 254 international medical graduates (IMGs) (2012–2019) from Zhejiang university were tracked in an investigation conducted in 2020, including 49 pursuing postgraduate programs, 123 passing medical license examination, and 13 being licensed in the United Kingdom, United States and Canada (37). A recent study showed 502 IMGs receiving primary medical qualifications in English-medium medical programs in Chinese universities are currently registered medical practitioners in the United Kingdom, 69.5% of whom are in staff grade and associate specialist (SAS) posts (38). To achieve a comprehensive and objective assessment of the MBBS program, it is imperative to conduct more extensive longitudinal studies tracking performance and outcomes across the stages from undergraduate students to health professionals.

In short, the above limited studies provided superficial insight into the multifaceted evaluation of the MBBS program in China, which has more than a dozen years of history, and helped to extract clues about the challenges related to improving program performance in the context of the IoME.

## 4 Challenges to the MBBS program

Challenges to the MBBS program emerged from student, curriculum and faculty dimensions in less than 2 decades.

Firstly, there is no unified comprehensive evaluation mechanism for student enrollment among HEIs (36), and the lower entrance requirements in some Chinese HEIs are preferred by IMSs, such as Indian students (4, 36). IMSs are enrolled based on their high school transcripts as proof of academic ability (36, 39, 40) and English proficiency for applicants from non-English-speaking countries, e.g., various demands on minimum scores of IELTS (5.5–7.5) or TOEFL (70–90), whereas interviews and/or entrance examinations are not compulsory and depend on the choice of each individual HEI. Typically, international applicants for undergraduate medical programs in the United Kingdom are required to achieve an A-level of AAA and a minimum IELTS score of 7.0 and attain the requisite scores on the admission test (e.g., University Clinical Aptitude Test, UCAT). Furthermore, performance in interviews and the quality of personal statements are also critically important components of the application process in the United Kingdom.

A study at a Guangdong-based university demonstrated that Chinese freshmen had considerably greater mathematical competence than their overseas MBBS counterparts (41), reflecting the need for stricter standard for IMS enrollment. Most of the IMSs are nonnative English speakers with a weak command of medical Chinese as well, inhibiting effective communication between IMSs, teachers and local patients and being recognized as the major and most common impediment to academic performance. Besides linguistic barriers, some IMSs experience well-being challenges (e.g., homesickness, loneliness, academic burnout, and stress) and cultural challenges (culture shock, e.g., traditions, customs, religion, and food) (36, 40).

Secondly, there are multiple challenges in the internationalization of medical curricula at the staff and institutional levels worldwide (42). The majority of medical schools in China use the discipline-based curriculum model, while curricular reforms are advocated in some medical schools, such as adopting the problem-based curriculum model, the organ-system-based curriculum model, and the clinical presentation-based curriculum model (43). According to MOE regulation, Chinese HEIs could make proper adjustments to the MBBS curriculum to tailor to the IMS countries of origin by adding or dropping courses or redistributing class hours in favor of certain subjects (26). Nevertheless, some IMSs perceived differences between the MBBS curriculum in China and in their home countries, a very tight course schedule, late exposure in clinical clerkships and limited clinical learning (36), or a lack of integration between basic sciences and clinical practice (13).

Thirdly, a couple of challenges also emerge from the perspective of faculties, such as insufficient English proficiency and pedagogical skills. A few teachers do not have adequate English skills for interaction with students of culturally diverse origin, and mainly rely on PowerPoints in their instructions without exhaustive explanation (44). During the 2018–2019 academic year, 14.6% of Chinese institutions implemented problem-based learning in medical education (7). Not all teachers are suitably qualified to teach organ-system-based courses and should acquire greater proficiency through collaborative lesson planning and trial lecturing (45).

In brief, the MBBS program encounters a series of challenges related to student, curriculum and faculty aspects in the context of the IoME. Each institution should prioritize those barriers and set up a tailor-made scheme for continuous quality improvement.

## 5 Actionable recommendations to address the challenges in the MBBS program in China

To address the multifaceted challenges in the MBBS program outlined above, several targeted recommendations emerge.

### 5.1 Students selection and support

The entrance exams, such as the Biomedical Admissions Test (BMAT), University Clinical Aptitude Test (UCAT), and International Students Admissions Test (ISAT), are an important part of the admission process for the MBBS programs in countries like the United Kingdom and Australia. These exams assess candidates’ potential to succeed in academic challenges and provide a useful reference for China. HEIs in China are entitled to conduct eligibility reviews, entrance examinations, or assessments for applicants to ensure the quality of admitted students (26). It is recommended that these HEIs should establish a consortium to manage an entrance test, which is used as one component of the admission decision in conjunction with other supporting information, such as interviewing, transcripts, certificates of high school, and English language proficiency test scores. Such a collaborative effort would ensure consistency in the evaluation process, enhancing the overall quality and integrity of the admissions procedure.

HEIs should provide adequate student support and counseling services, including supplementary tuition, the development of learning skills, and personal support for cultural, emotional, and stress-related problems (27, 29). A strategy for cross-sectional and longitudinal surveys should be developed to investigate program performance, yield insight, and support for career growth after graduation.

### 5.2 Curriculum integration

The standards for basic medical education in China (the 2022 revision) acknowledge both the discipline-based curriculum model and various integrated curriculum models (46). Horizontal integration (multidisciplinary and interdisciplinary integration) and vertical integration (integration from basic sciences to clinical sciences) are advocated by the standards and are regarded as developmental goals that align with the high standards of undergraduate clinical medical education promoted internationally. The extent to which the developmental goals are achieved can vary widely among medical schools, depending on their stage of development, available resources, and educational policies (46). The national standards body for medical education should design a roadmap and guideline to expedite the integration of medical curricula in batches among medical schools, as the advanced educational resources, such as smart classrooms, medical AR and VR setups, and human patient simulators, are increasingly becoming prevalent in Chinese medical schools. Furthermore, research projects focused on integrating curriculum reforms should be initiated at both the university and national levels. It is essential to collect insights from students, faculty, and employers regarding the effectiveness of the integrated curriculum models, with their feedback driving continuous enhancements.

### 5.3 Faculty improvement

Professional development programs (PDPs) and both ‘inbound’ and ‘outbound’ strategies could be adopted to improve the faculty’s teaching capabilities. PDPs, such as mentorship programs, English proficiency improvement program, peer observation and collaboration, and personalized professional growth plans, could be implemented for quality improvement. Since 2001, attracting overseas-educated talents to return and work in China has been an important national strategy (47). Since then, the proportion of medical school faculty with overseas study experience has steadily increased. Overall, these teachers are well-versed in integrated curriculum teaching approaches and demonstrate strong English communication skills; thus, they should be encouraged to actively engage in and assume responsibilities for teaching integrated courses in the MBBS program. Another complementary approach involves selecting teachers to participate in faculty development programs at overseas medical institutions, providing them with international exposure and advanced educational experiences.

The effectiveness of the implemented improvement suggestions should be monitored and evaluated using an instrument developed in accordance with national and international medical education standards. This instrument should cover both process measures (e.g., alignment of course learning objectives, instruction quality) and outcome measures (e.g., residency matching results, medical licensing exam results). The performance of the MBBS program should also be accredited by the World Federation for Medical Education (WFME) recognized agencies.

## 6 Conclusion

The global needs-based shortage of physicians is expected to reach 2.3 million by 2030, with the most significant deficits in Africa, which is suffering a shortfall of 1.1 million, and Southeast Asia, grappling with a gap of 1 million (48). The IoME plays a role in providing a health workforce for transformative work that crosses national boundaries (49). Compared with developed countries in Europe and America, China is a new competitor and provider of international medical education.

The IoME was ushered in by the process of the IoHE, which was concurrent with the Chinese open-up policy in the late 1970s. Chinese HEIs actively and responsively engaged in government-led IoHE schemes and strived to improve their positions in global university rankings and league tables. The concomitant development of the MBBS program, an example of the IoME in China, was achieved on the basis of sustained policy support. Assessments of the program based on the existing scholarly literature and research could be linked to the challenges arising from students, curricula and faculty that constitute the core of 3-dimensional model of the IoME. Higher linguistic and academic admission thresholds, adequate student support services, international alignment of the medical curriculum to cater to students’ diversity, and improvement in teachers’ English proficiency and pedagogical skills are necessary for enhancing the quality of the MBBS program. These improvements are conducive to building a brand project of the IoME in China and alleviating medical workforce shortages in regional and global contexts. It is anticipated that Chinese HEIs will engage more proactively in exploring the IoME, with the level of performance steadily advancing on the basis of continuous accumulation of experience and effort.
